# Developmental Exposure to Estrogen Alters Differentiation and Epigenetic Programming in a Human Fetal Prostate Xenograft Model

**DOI:** 10.1371/journal.pone.0122290

**Published:** 2015-03-23

**Authors:** Camelia M. Saffarini, Elizabeth V. McDonnell-Clark, Ali Amin, Susan M. Huse, Kim Boekelheide

**Affiliations:** 1 Department of Pathology and Laboratory Medicine, Brown University, Providence, Rhode Island, United States of America; 2 Department of Pathology and Laboratory Medicine, Rhode Island Hospital, Providence, Rhode Island, United States of America; University of Hyderabad, INDIA

## Abstract

Prostate cancer is the most frequent non-cutaneous malignancy in men. There is strong evidence in rodents that neonatal estrogen exposure plays a role in the development of this disease. However, there is little information regarding the effects of estrogen in human fetal prostate tissue. This study explored early life estrogen exposure, with and without a secondary estrogen and testosterone treatment in a human fetal prostate xenograft model. Histopathological lesions, proliferation, and serum hormone levels were evaluated at 7, 30, 90, and 200-day time-points after xenografting. The expression of 40 key genes involved in prostatic glandular and stromal growth, cell-cycle progression, apoptosis, hormone receptors and tumor suppressors was evaluated using a custom PCR array. Epigenome-wide analysis of DNA methylation was performed on whole tissue, and laser capture-microdissection (LCM) isolated epithelial and stromal compartments of 200-day prostate xenografts. Combined initial plus secondary estrogenic exposures had the most severe tissue changes as revealed by the presence of hyperplastic glands at day 200. Gene expression changes corresponded with the cellular events in the KEGG prostate cancer pathway, indicating that initial plus secondary exposure to estrogen altered the PI3K-Akt signaling pathway, ultimately resulting in apoptosis inhibition and an increase in cell cycle progression. DNA methylation revealed that differentially methylated CpG sites significantly predominate in the stromal compartment as a result of estrogen-treatment, thereby providing new targets for future investigation. By using human fetal prostate tissue and eliminating the need for species extrapolation, this study provides novel insights into the gene expression and epigenetic effects related to prostate carcinogenesis following early life estrogen exposure.

## Introduction

Prostate adenocarcinoma (PCa) is the most frequent non-cutaneous cancer in men, as well as the second leading cause of male cancer-related death in the United States. In 2013, an estimated 238,000 men developed this disease, resulting in 29,000 deaths [1]. There is strong evidence that both diet and exposure to exogenous estrogens play an influential role in the formation of PCa [2–5]. Exogenous estrogens act as endocrine disrupting chemicals and have the ability to hinder the ability of the natural hormone to function properly [6]. Xenoestrogens are ubiquitous in the environment and can be found in food (phytoestrogens) and packaging materials (e.g. plastic and polystyrene). In particular, phytoestrogens are a potential concern in baby formula [7]. The phenotypic progression of this disease involves the transformation from a normal epithelial state to hyperplasia, prostatic intraepithelial neoplasia (PIN), and subsequent aggressive invasive adenocarcinoma [8]. While PCa is widely known to afflict older men, PIN or high-grade PIN (HGPIN) have been identified in autopsies of 20–30 year old men [8, 9], suggesting that the disease may begin much earlier.

Rodent studies have shown the fetal and perinatal periods to be highly susceptible to endocrine disruption. Developmental exposures to low-dose estradiol [10] or bisphenol A (BPA) can have long-lasting pathologic effects in rat prostate, resulting in hyperplasia and pre-malignant lesions [11]. The “one-hit” versus “two-hit” rodent model was established to explore developmental estrogenization [12] by exposing neonatal rat pups to estrogen soon after birth (“one-hit”), with or without a subsequent secondary later-life exposure (“two-hit”) [13]. Early exposure to high-dose estradiol in this model results in lasting prostate pathological changes in the form of inflammation, hyperplasia, and PIN [10, 13]. Interestingly, the rate of PIN increases dramatically when early high-dose estradiol is combined with later-life exposure to estradiol [11, 13, 14]. This rodent model supports the “hormonal imprinting” hypothesis [15] that describes the priming effects of early life exposure to estrogenic compounds on prostate tissue, rendering it vulnerable to gene modifications and thereby making it more susceptible to developing prostatic neoplasms.

Rodent data from the “one-hit” versus “two-hit” model provides compelling evidence for a similar process in humans, where early acute perinatal exposure to estrogens followed by chronic low-dose exposures in adulthood is not uncommon. Evidence of PIN lesions provides strong warning signs of subsequent aggressive carcinoma in men as young as 20 years old [8, 9], suggests a role for early life disruption in PCa commonly arising in older men. This mechanism is unknown, but current studies are looking at changes in epigenetic processes such as DNA methylation; epigenetic alterations have the potential to transform progenitor cell development, producing differentiation defects leading to the early onset of PCa [14].

The previous rodent model of developmental estrogenization has been informative and hypothesis-generating but ultimately is insufficient to report on the fetal origins of human PCa. Important morphological and hormonal differences between the rodent and human prostate [16] require studies using human fetal tissue to confirm a similar developmental estrogenization pattern. Estrogens are ubiquitous in the environment [5, 7] and it is not surprising that early exogenous exposure could lead to long-lasting estrogen-related developmental effects on both maturation and growth in this hormonally-sensitive gland. The present study explores human developmental estrogenization using a similar “one-hit” versus “two-hit” model. Human fetal tissue is used to eliminate the need for species extrapolation and facilitate the broader goal of understanding the fetal origins of PCa.

## Materials and Methods

Brown University entered into an IRB Intra-Agency Agreement (#10–15) naming Women and Infants Hospital as the IRB of record for this study (project #: 09-09077; protocol title: Children's Environmental Health Project). Informed and written consent was obtained from donors. Brown University IACUC reviewed and approved all experimental protocols (#1211990036) for the project entitled, "Formative Center for the Evaluation of Environmental Impacts on Fetal Development." Isoflurane was used for both anesthesia and euthanasia. All animals were euthanized by an overdose of isoflurane (Baxter Healthcare Corporation, Deerfield, IL) followed by cervical dislocation.

### Chemicals

Corn oil (CAS#: 8001-30-7), estradiol benzoate (β-estradiol 3-benzoate, CAS#: 50-50-0), and testosterone (CAS#: 58-22-0) were obtained from Sigma Aldrich (St. Louis, MO). Placebo pellets (SC-111) and 2.5 mg pellets of β-estradiol 3-benzoate (SE-281) were obtained from Innovative Research of America (Sarasota, FL).

### Silastic Capsule Preparation

Silastic lab tubing (Catalog number: 508-006, Dow Corning, Midland, MI) with an inner diameter of 1.47 mm and outer diameter of 1.96 mm, was cut into 2-cm pieces and weighed. Silastic tubing was then filled with testosterone (T1500, Sigma) and weighed again to obtain the final dose of 25 mg testosterone within each tube. Tubing was then sealed at both ends with the medical adhesive, acetoxy silicone (A-564, Factor II Inc., Lakeside, AZ), and allowed to dry for at least an hour [17].

### Animals

Athymic nude male mice (Crl:NU-Foxn1^nu^, strain code 490, aged 43–56 days old) deficient in T cells were purchased from Charles River Laboratories (Wilmington, MA). Mice were kept in groups of six prior to surgery, and then divided into groups of two based upon treatment. Purina Rodent Chow 5001 (Farmer’s Exchange, Framingham, MA) and water was freely accessible. Rodent Chow 5001 is managed using Purina’s Constant Nutrition management system to reduce lot-to-lot variability and identify potentially disruptive environmental contaminants. Daily assays and subsequent refinements in formulation ensure that the chow is consistent over the lifetime of the animal [18]. In accordance with National Institutes of Health Regulations, the animals were housed in a temperature and humidity controlled room with a continuous alternating 12-hour light-dark cycle. Brown University Institutional Animal Care and Use Committee (IACUC) reviewed and approved all experimental protocols.

### Acquisition of Human Prostate Tissue

Human fetal prostate tissues from the 2^nd^ trimester (gestational ages 15–21.5 weeks) were acquired from Women and Infants Hospital (Providence, RI). This gestational age group was chosen for xenotransplantation because it is during this time that organogenesis occurs, and it is an important period of development for the fetal prostate [19]. A detailed description of human fetal tissue acquisition from spontaneous pregnancy losses, in accordance with the Institutional Review Board, is described in detail in De Paepe *et al* [20]. Briefly summarized, written and informed consent was obtained from the donors. Board certified pathologists evaluated the condition of the human tissue before xenotransplantation. Samples were excluded if macerated. To protect donor identity, personal information was replaced with anonymous identifiers for all human samples [20].

### Xenotransplantation Surgery

Surgeries were performed at the Xenotransplantation Core Facility of Brown University as described in Saffarini *et al*. [16], in accordance with Brown University’s Institutional Review Board requirements. Human tissue was kept in ice-cold transport media containing Leibovitz L-15, penicillin (100 U/mL), streptomycin (100 μg/mL), and gentamycin (50 μg/mL). Male mice (aged 50–65 days old) were placed under isoflurane anesthesia (Baxter Healthcare Corporation), and a peritoneal incision was made in the skin and muscle to expose the kidney. A small nick was made to expose the renal subcapsular space of the kidney, and a piece of human fetal prostate tissue (1–3 mm in size) was inserted. The insertion of prostate tissue under the renal subcapsular space has been well-documented and shown to be an optimal site for implantation [21]. The incision was then sutured and the skin was stapled. Animals were monitored daily for the following two weeks for adverse effects resulting from surgery (i.e. wound opening, malnutrition, etc.).

### Xenograft Dosing Paradigm

The dosing paradigm resembles the rodent “one-hit” versus “two-hit” model [13, 22]. Mice were given a subcutaneous injection of corn oil (control) or 250 μg/kg of β-estradiol 3-benzoate (treatment) in corn oil immediately following surgery, and then every other day for a total of 3 injections (“one-hit”). Previous studies have used 25 μg estradiol benzoate (“one-hit”) and found it to be highly bound to α-fetoprotein in neonatal serum. This dose also represents a previously-determined relevant exposure to diethylstilbestrol [23]. The current 250 μg/kg dose was chosen to elicit an effect on human prostate xenografts without causing toxicity to the rodent host. Xenografts were then collected at 7, 30, or 90 days to evaluate the effects of early estrogen exposure. At day 90, a subset of mouse hosts were given a slow-release extended dose of a 2.5 mg pellet containing β-estradiol 3-benzoate, which released estradiol at a rate of 0.042 mg/day for 110 days (“two-hit”). A supplementary treatment of 25 mg of testosterone was given with the estradiol as a slow-release 2-cm silastic capsule to maintain testosterone levels and prevent prostatic involution [13]. This combination of 2.5 mg of estradiol and 25 mg of testosterone has been successful in generating cancerous lesions in mice [24]. Control animals received the placebo pellet for β-estradiol 3-benzoate, and an empty silastic capsule. Capsules and pellets were replaced after 8 weeks in mouse hosts, and xenografts were collected on day 200 (**S1 Fig.**).

Human tissue (gestational ages 15–21.5 weeks) that originated from 10 samples was distributed across the treatment groups (15 weeks; n = 1, 16 weeks; n = 1, 20 weeks; n = 6, 21 weeks; n = 1, 21.5 weeks; n = 1). The majority of the samples collected originated at 20 weeks (n = 6), so all treatment groups had at least n = 2 with samples originating at 20 weeks. Samples that originated at 15, 16, and 21.5 weeks (n = 1 each group) was distributed across groups so that at least one of these was in each of the time-points and was not dominating a particular treatment or group.

### Serum Collection and Hormone Analysis

Blood was collected from mouse hosts via cardiac puncture at the time of euthanasia using isoflurane anesthesia (Baxter Healthcare Corporation). Needles (26 gauge x 5/8) were used to collect mouse host blood (BD, Franklin Lakes, NJ) before placement in serum separator tubes (BD). Blood was allowed to coagulate for 30 minutes prior to centrifugation at 5600 x g for 12 minutes, and serum was placed into 1.5 mL tubes. Serum was stored at −80°C until sample processing at University of Virginia Center for Reproduction Ligand Assay and Analysis Core (Charlottesville, VA). Assays for estradiol, testosterone, and luteinizing hormone/follicular-stimulating hormone (LH/FSH) multiplex were performed and estradiol was significantly increased as expected in the 7-day estrogen-treated groups as well as the 200-day C/E and E/E treatment groups (**S2 Fig.**).

### Xenograft Collection and Histological Processing

Xenografts were harvested at 7, 30, 90, and 200 days post-surgery. The prostate implants were divided into four pieces. One piece was fixed in 10% neutral buffered formalin for paraffin processing, and a second was placed in Tissue-Tek O.C.T. compound (Electron Microscopy Sciences, Hatfield, PA) and placed in liquid nitrogen for later use in laser capture microdissection. Of the remaining two pieces, one was used for DNA extraction and the other was homogenized in Trizol for RNA extraction; both samples were snap frozen in liquid nitrogen and stored at −80°C until further use. Paraffin sections were cut at 5 μm, and slides were stained with hematoxylin and eosin. Slides were scanned into an Aperio Scanscope CS microscope (Aperio Technologies, Vista, CA) and histological analysis was executed using ImageScope software (Aperio Technologies).

### DNA and RNA Isolation

DNA was isolated using the QIAamp DNA Micro Kit (Qiagen, Valencia, CA), while RNA was isolated using the RNeasy Micro Kit (Qiagen) according to manufacturer’s instructions. DNA was eluted in DNA suspension buffer (Teknova, Hollister, CA). RNA isolation used the additional recommended DNase step provided in the kit.

### Immunohistochemistry

Prostate xenografts were stained for p63 (Santa Cruz Biotechnology Inc., Santa Cruz, CA, sc-8431, 0.5 μg/ml), prostate specific antigen (Dako Cytomation, Carpinteria, CA, A0562, 0.2 μg/ml), and caldesmon (Sigma-Aldrich, St. Louis, MO, C4562, 2 μg/ml) as previously described [16]. Briefly, 5 μm paraffin tissue sections were de-paraffinized and rehydrated prior to staining. Antigen retrieval was performed using a 10mM citrate buffer (pH 6) and a vegetable steamer for 20 minutes. Avidin and biotin was blocked (SP-2001, Vector Laboratories, Burlingame, CA), and antibodies were stained overnight at 4 degrees Celsius. The appropriate secondary antibody was used, and an avidin-biotin complex (PK-6100, Vector Labs) and peroxidase substrate 3, 3’-diaminobenzidine (Vector labs) method was used for visualization. A negative control (containing no primary antibody) was performed for each experiment.

### Ki-67 Quantification

Tissue sections were stained with Ki-67 antibody (Dako Cytomation, Carpinteria, CA, M7240, 0.5 μg/ml) as previously reported [16]. The IHC Nuclear Macro (Aperio) was used to measure the total number of nuclei found within the epithelial and stromal compartments of prostate xenografts. Positive nuclei, as defined by dark punctate nuclear staining, were counted in the epithelial and stromal compartments with sample identity (treatment and age) blinded to the scorer. Area (mm^2^) of each xenograft compartment was measured, and the Ki-67 proliferative index (number of positive nuclei divided over the total number of nuclei) was determined in both compartments. Statistical analyses were performed using a student’s T-test with p-value significance (p-value<0.01).

### Laser Capture Microdissection (LCM)

Arcturus XT Laser Capture Microdissection system (Applied Biosystems, Grand Island, NY) was used to isolate the epithelial and stromal tissue compartments of the human prostate xenografts. Tissue embedded in O.C.T. medium was cut at 7 μm on the cryostat (Leica Microsystems Inc., Buffalo Grove, IL) and placed onto polyethylene naphthalate (PEN) membrane slides (Applied Biosystems). Slides were stored at −80°C until microdissection processing. Tissue sections were processed, rehydrated, and stained according to manufacturer’s instructions. Epithelial tissue was captured using the infrared system option and collection of stromal tissue immediately followed using the ultra-violet laser system. Isolated tissue was captured on Arcturus Capsure Macro LCM Caps (Applied Biosystems) and DNA was isolated from caps using the QIAamp DNA Micro Kit (Qiagen).

### PCR Array

A custom SABiosciences PCR array (Qiagen) was created incorporating 40 genes generated from primary literature that evaluated prostate ductal and mesenchymal growth, cell survival, tumor growth, and androgen biosynthesis (**S1 Table**). Each PCR plate contained 3 internal controls to detect genomic DNA contamination (GDC), synthetic DNA (PPC), and synthetic RNA (RTC). Five housekeeping genes were chosen (18SrRNA, GAPDH, TBP, B2M, and RPLP1). Samples were prepared according to manufacturer’s instructions, and each treatment group was divided between the plates ensuring that each plate contained a variety of different time-points. Samples were placed onto 384-well plates using an epMotion 5075 automated pipetting system (Eppendorf, Hauppauge, NY). Plates were run using a ViiA 7 Real-Time PCR System (Life Technologies, Grand Island, NY) using cycling conditions recommended by the manufacturer.

### DNA Methylation Array

Genomic DNA from whole tissue samples and LCM-isolated epithelial and stromal prostate tissue were sent to Yale’s Center for Genome Analysis (Yale School of Medicine, West Haven, CT). For these experiments, human fetal tissue prior to implantation was gestational age 16–21.5 weeks old (200 day control/control; n = 3, 200 day estrogen/estrogen; n = 4). These samples were further processed using the Infinium HumanMethylation450 BeadChip Assay (Illumina, Inc., San Diego, CA), which included bisulfate conversion, according to manufacturer’s protocol. Methylation status of >480,000 CpGs was analyzed.

### Statistical Analysis and visualization

Statistical analysis and figures for Ki-67 quantification (Student’s t-test) and hormone analysis (ANOVA with Bonferonni correction) data were created using GraphPad Prism (La Jolla, CA). A treatment group is described as the original piece of human fetal prostate representing the statistical unit for comparison across the different treatments. 7-day control: n = 3; 7-day estrogen exposure: n = 2; 30-day control: n = 3; 30-day estrogen exposure: n = 4; 90-day control: n = 4; 90-day estrogen exposure: n = 5; 200-day control/control: n = 4; 200 day estrogen/control: n = 4; 200-day control/estrogen: n = 2; 200-day estrogen/estrogen: n = 4.

Raw qPCR data were imported into the R statistical environment. Data were normalized against the housekeeping genes and ddCt values were calculated using the SLqPCR package [25]. Significant fold changes in gene expression were evaluated using the limma package [26] on the log2 transformed values. The DNA methylation array data were normalized using the subset-quantile within array normalization method [27] (R package: minfi, http://www.bioconductor.org/packages/release/bioc/html/minfi.html [28]) and batch-corrected using ComBat (R package sva [29]) adjusting for both treatment and tissue type. CpG locations altered by estrogen treatment were identified using a linear model on the logit-transformed methylation values and a false discovery rate threshold of 0.05 on each tissue type separately (epithelium, stroma) or combined (whole tissue) (R command dmpFinder in minfi). Annotations were assigned to each location using University of California Santa Cruz (UCSC) gene names information as provided in the IMA package [30]. Gene enrichment analysis was performed using the functional annotation cluster tool on the DAVID website [31].

## Results

### Estrogen treatment resulted in marked histopathologic changes in prostate xenografts

**Fig 1:**
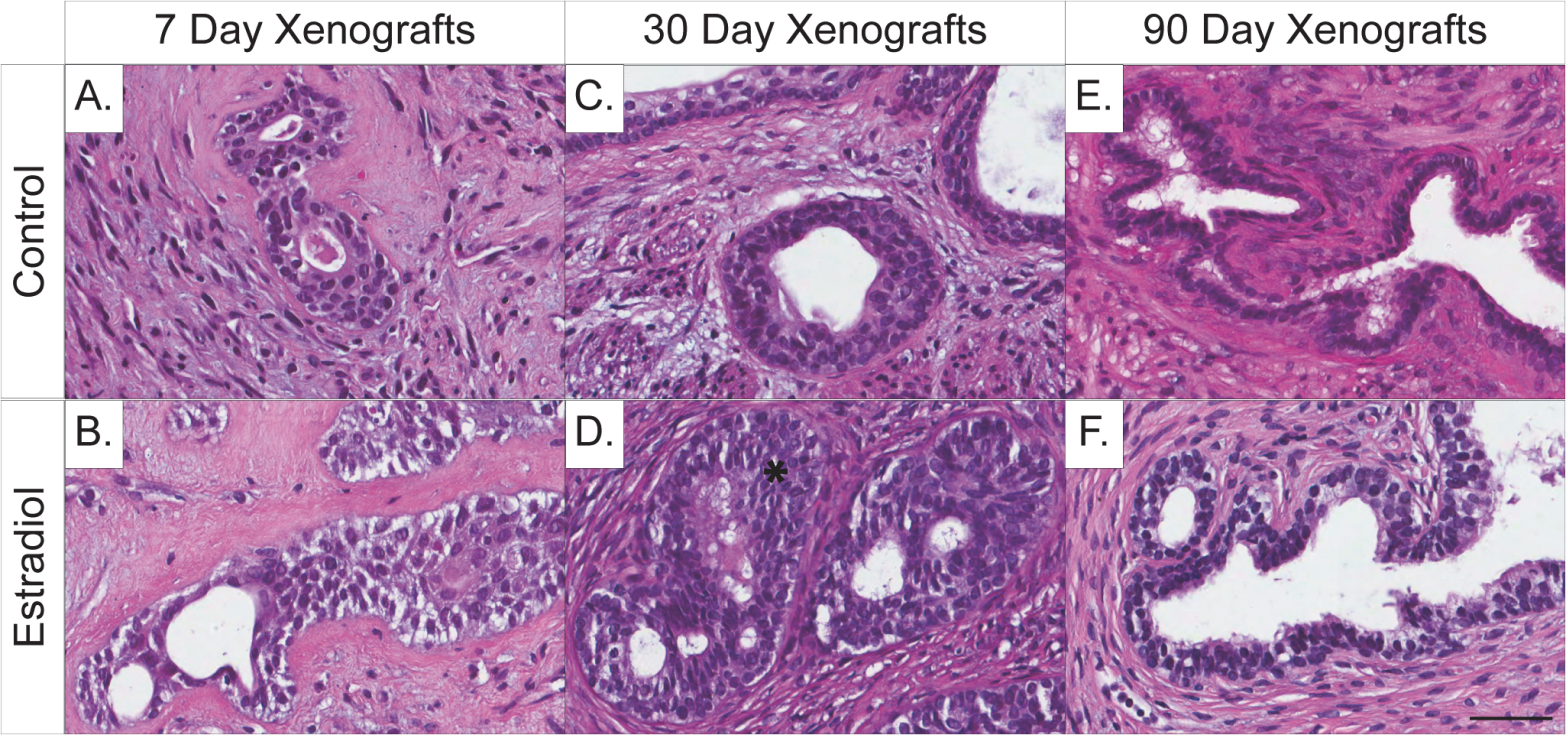
Histology of human prostate xenografts collected at 7, 30, and 90 days, demonstrating phenotypic tissue changes after a single estradiol dose in the “one-hit” dosing paradigm. (**A**) At 7 days post-implantation, control xenografts display a cellular mesenchyme and small primordial glands, which is representative of normal human fetal tissue. (**B**) Estrogen-treated xenograft at 7 days post-implantation demonstrates minor ductal branching with some vacuolization. (**C**) At 30 days post-implantation, control xenografts present with both well-developed glands and stroma. (**D**) Estrogen-treated xenografts at 30 days display significant basal cell hyperplasia (*) encompassed by an un-developed mesenchyme. (**E-F**) At 90 days, both control- and estrogen-treated xenografts have well-developed tortuous glands with no visible differences between control and treated. (**A-F**) Gestational age of human fetal prostate prior to implantation is 20 weeks. (Hematoxylin and Eosin staining; scale bar = 50 μm).

Histopathological analysis of the 7, 30, and 90 day (“one-hit”) prostate xenografts (20 weeks gestation) showed differences between control (**Fig. 1A,C, &E**) and treated (**Fig. 1 B, D, &F**) groups in both epithelial and stromal compartments. Canonical markers for estrogen receptor alpha and beta have been assessed, and are present in human prostate xenografts at these early time-points, as shown by our previous publication [16]. Interestingly, these receptors did not demonstrate any gene expression changes in the PCR array analysis (**S2 Table**). The 7-day control xenografts were immature, with a cellular mesenchyme and small primordial glands (**Fig. 1A**). In comparison, 7-day estradiol benzoate-treated samples had small glands, along with minor vacuolization in the stroma. The estrogenized stroma was less cellular than the control (**Fig. 1B**). In both control and estradiol benzoate-treated 7-day xenografts, the glandular basal cells were found layered on top of each other, a condition referred to as basal cell hyperplasia, providing further evidence that the tissue at 7-days post-xenotransplant is still phenotypically fetal and adjusting to the adult mouse host hormonal environment (**Fig. 1A&B**). Measurements of estradiol in the 7-day estrogen-treated hosts demonstrated a significant increase in the serum estradiol levels averaging approximately 200 pg/mL (**S2 Fig.**). This dose is physiologically relevant as this is similar to the fluctuating high levels of estradiol seen in women during pregnancy [32, 33].

Control xenografts at 30 days were starting to differentiate, indicated by reduced basal cell hyperplasia, well-developed and functional prostate glands containing secretions, and well-developed stroma containing both smooth muscle cells and fibroblasts (**Fig. 1C**). In contrast, estradiol benzoate-treated 30-day xenografts displayed small rudimentary glands with a significant amount of basal cell hyperplasia and an under-developed immature cellular stroma. Limited secretory function appeared to be present in these small prostate glands (**Fig. 1D**). The 90-day control and estradiol benzoate-treated xenografts displayed similar histopathology, with established tortuous glands that had secretions present and was surrounded by a mature stroma (**Fig. 1E&F**).

**Fig 2:**
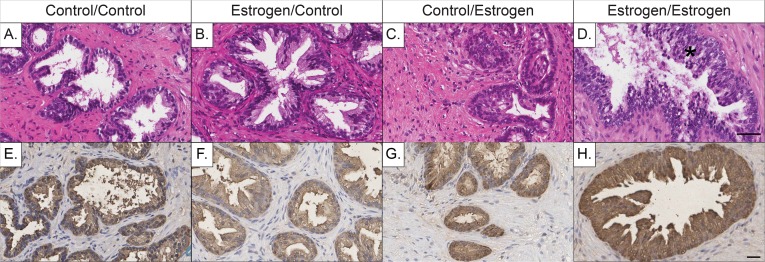
Histology and prostate specific antigen (PSA) staining of 200-day human prostate xenografts exposed to varying initial (corn-oil or estradiol) and later-life (control placebo capsules or estrogen pellets and testosterone capsules) exposures. (**A**) Control human prostate xenografts (control/control) contained phenotypically normal prostatic ducts surrounded by mature stromal cells, (**E**) with proper PSA staining in the luminal cells of ducts. (**B**) Human prostate xenografts given an initial treatment of estrogen, with no subsequent treatment (estrogen/control) demonstrated a mature phenotype containing both nicely developed glands and a normal cellular stroma, (**F**) with normal PSA staining similar to C/C-treated xenografts. (**C**) In comparison, xenografts given only a secondary treatment of estrogen and testosterone (control/estrogen), appeared to have areas of immature or undeveloped tissue (**G**) with a greater amount of PSA staining in the lumen compared to C/C-xenografts. (**D**) An initial and secondary exposure to estrogen (estrogen/estrogen) exhibited an extensive amount of glandular hyperplasia (*), (**H**) as well as a significant amount of PSA staining. Figure headings represent treatment conditions as initial/secondary exposure. (**A-H**) Gestational age of human fetal prostate before implantation is 20 weeks. (Hematoxylin and Eosin staining and counter-stain; scale bar = 50 μm).

After evaluation of human xenografts at early time-points, later-life treatment effects resulting from an initial and secondary treatment of control or estradiol benzoate were investigated at 200 days (**Fig. 2**). Xenografts given no initial or secondary treatment (C/C) presented with an adult phenotype that consisted of well-developed glands with secretions present within the lumen that was surrounded by mature stromal cells. Some atrophic glands were present, likely due to the large size of the implant, with the mouse kidney capsule acting as a restraint and preventing the xenograft from expanding. This atrophy may also result from the inability of the glandular secretions to exit the prostate (**Fig. 2A**). When estradiol benzoate treatment was administered initially without subsequent estrogen exposure (E/C), xenografts exhibited well-developed glands surrounded by a cellular, mature stroma similar to C/C-xenografts. With the exception of some mild hyperplasia, this treatment group looked phenotypically normal, similar to normal adult prostate (**Fig. 2B**). Xenografts that were given no initial treatment but only a secondary treatment of estrogen (C/E), contained epithelial cells with a proper basal and luminal layer, albeit some areas of the xenograft had immature or undeveloped ducts. These areas of immature tissue did not appear to affect secretory activity compared to C/C-xenografts. In addition, while there was significant stromal development present, in some areas the stroma was immature and underdeveloped (**Fig. 2C**). Xenografts given both an initial and secondary (“two-hit”) exposure of estradiol benzoate (E/E) showed predominantly hyperplasia of the luminal cells of prostatic glands, and serum levels indicated a significant increase in estradiol in mouse hosts (**S2 Fig.**). Stroma in this group was well-developed with abundant smooth muscle and fibroconnective tissue without any evidence of stromal hyperplasia (**Fig. 2D**).

Staining for the functional prostate specific antigen (PSA) marker was performed to investigate the effects of estrogen treatment on ductal secretion [34]. Xenografts given C/C- and E/C-treatment had normal staining of the ducts and surrounding stroma, with the most intense positivity occurring at the apical surface of the ducts (**Fig. 2E-F)**. Xenografts given C/E-treatment demonstrated a similar pattern with somewhat stronger PSA staining compared to the C/C-treated xenografts (**Fig. 2G**). In contrast, xenografts that were given E/E-treatment had an increased intensity of PSA staining in hyperplastic ducts, with a uniform intensity of the signal in the basal and apical regions of the ductal cells. Additionally, staining was stronger in the surrounding stroma compared to the other treatment groups (**Fig. 2H**).

### Estrogen treatment resulted in morphological changes at the early time-points

**Fig 3:**
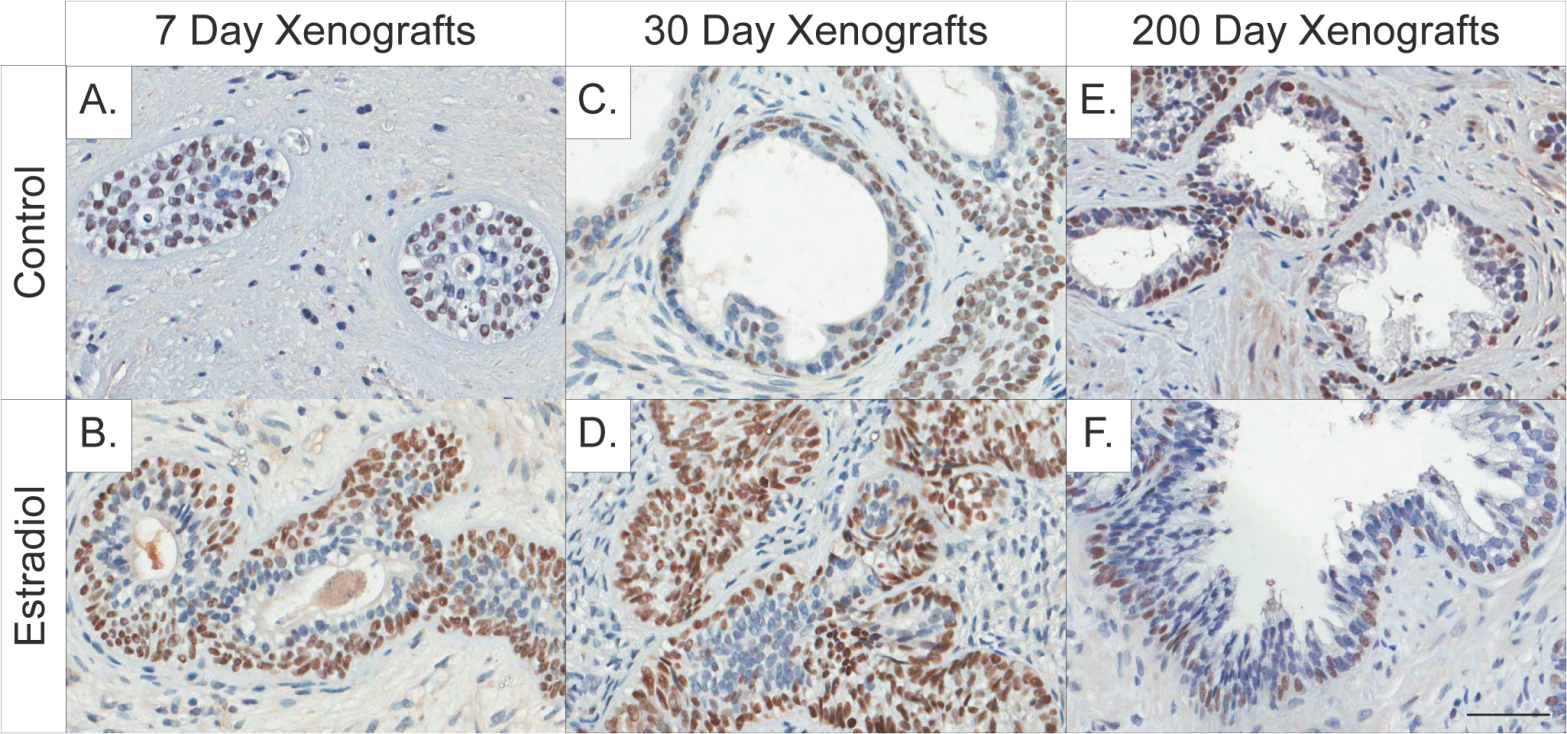
Immunohistochemical staining of the epithelial basal cell marker p63 in 7, 30, and 200-day control and estrogen-treated human prostate xenografts. Basal cell hyperplasia is evident in both (**A**) control and (**B**) estrogen-treated 7-day xenografts. At 30 days post-implantation basal cell hyperplasia is reduced in (**C**) control xenografts but is still prominent in the (**D**) estrogen-treated xenografts. At 200-days post-implantation, (**E**) control/control xenografts appear phenotypically mature with p63 lining the basal cell layer of adult prostatic ducts. Similarly, (**F**) the prostatic basal cells appear mature in 200-day estrogen/estrogen-treated xenografts while the luminal cells appear hyperplastic. (**A-F**) Gestational age of human fetal prostate before implantation is 20 weeks. (Hematoxylin counterstain; scale bar = 50 μm).

Further evaluation of prostate maturation was assessed using the epithelial basal cell marker p63 and the stromal adult muscle marker caldesmon. Basal cell hyperplasia was present in the 7-day xenografts in both control and estradiol-benzoate treated xenografts as confirmed by the strong nuclear p63 staining, albeit the estrogen-treated xenograft appeared with a slightly stronger and more punctate nuclear p63 staining (**Fig. 3A&B**). At 30-days post-implantation, basal cell hyperplasia was significantly reduced in the control xenografts (**Fig. 3C**), but persisted in estradiol-treated xenografts (**Fig. 3D**). By 200-days post-implantation, C/C xenografts presented with a normal mature phenotype, with p63 lining the basal lamina within the mature prostatic glands (**Fig. 3E**). Additionally, basal cell hyperplasia was not present in prostatic glands in 200-day E/E-treated xenografts (**Fig. 3F**).

**Fig 4:**
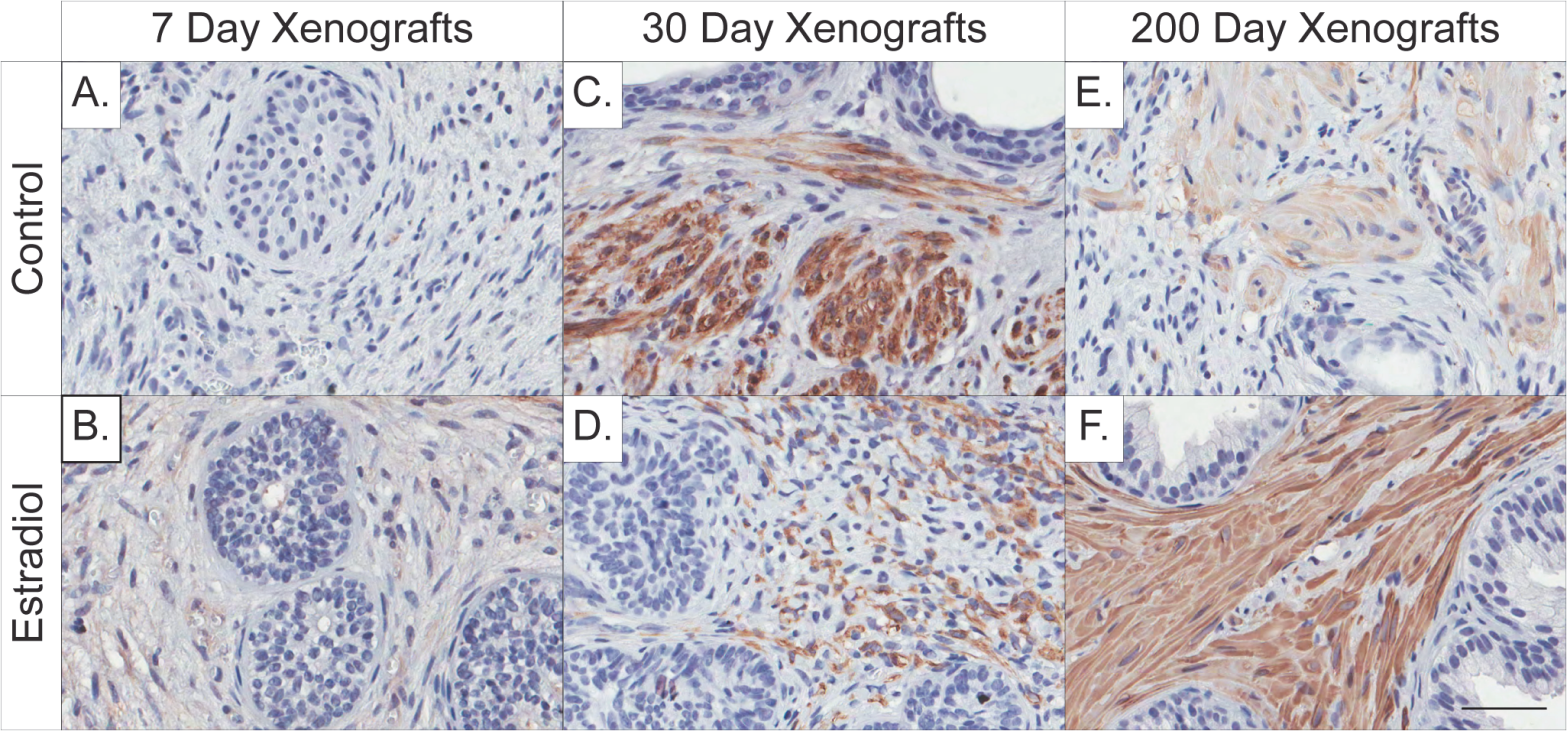
Immunohistochemical staining of the adult stromal muscle marker caldesmon in 7, 30, and 200-day control and estrogen-treated human prostate xenografts. Caldesmon staining is minimally present in both 7-day (**A**) control and (**B**) estrogen-treated xenografts, as these both present with a phenotypically fetal stromal environment. At 30-days, (**C**) control xenografts stain strongly for caldesmon and display a more mature stromal environment and the presence of smooth muscle bundles. In comparison, (**D**) the 30-day estrogen-treated xenografts stain for caldesmon with an immature muscle structure compared to control. At 200-days, (**E&F**) both control and estrogen-treated xenografts display a significant amount of caldesmon staining indicative of normal adult stromal tissue. Gestational age of human fetal prostate before implantation is 20 weeks. (Hematoxylin counterstain; scale bar = 50 μm).

In addition to epithelial maturation, stromal maturation was also assessed in prostate xenografts using the adult muscle marker caldesmon. Caldesmon was minimally present in both 7-day control (**Fig. 4A**) and estradiol-treated (**Fig. 4B**) xenografts. Stromal tissue appeared more phenotypically mature in 30-day control xenografts (**Fig. 4C**), with intense caldesmon staining. Staining was also present in 30-day estradiol-treated xenografts (**Fig. 4D**), but was found in stroma that was underdeveloped, immature, and highly cellular. By 200-days post-implantation, both control and estradiol-treated xenografts stained strongly for caldesmon in phenotypically mature prostate tissue (**Fig. 4E&F**).

### Initial and secondary estrogen exposure (E/E) increased epithelial cell proliferation

**Fig 5:**
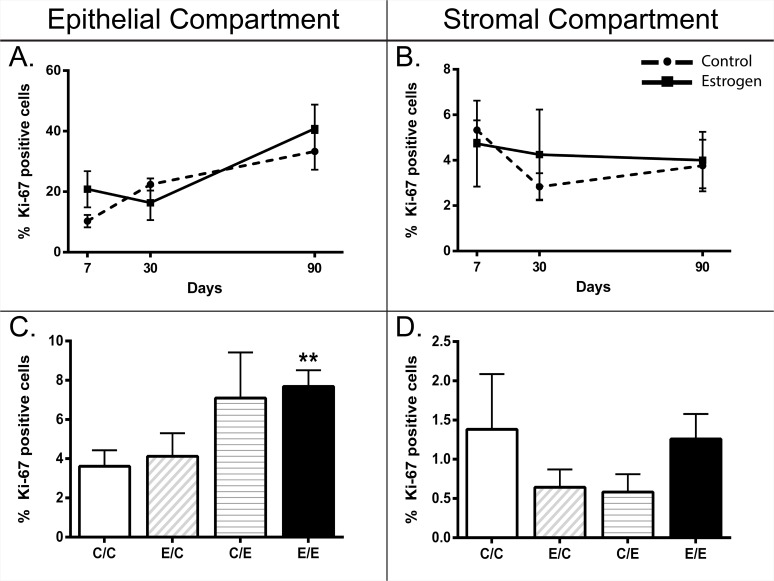
Ki-67 proliferative index in both epithelial and stromal compartments of human prostate xenografts at 7, 30, 90, and 200 days post-implantation. While an increasing percent of epithelial cells is proliferating over time, estrogen treatment does not affect prostate xenograft growth in the 7, 30 or 90-day xenografts in either the (**A**) epithelial or (**B**) stromal compartments. At 200 days post-implantation (**C**) there is a significant increase in epithelial proliferation in xenografts given an initial and secondary treatment of estrogen compared to control, (**D**) while there was no significant change in the stromal compartment. (**C-D**) The x-axis on the 200-day bar graphs are depicted as initial/secondary treatment in which C = control, and E = estrogen treatment. Ki-67 positive nuclei were measured as a percent of the total number of nuclei in each respective compartment. Line and bar graphs indicate the mean % Ki-67 positive cells ± SEM, ** indicates t-test significance relative to control of p<0.01. Legend: —●— Control, -■- Estrogen. Age of human fetal prostate prior to implantation was 15–21.5 weeks gestation. Sample size (n) for each group is as follows: 7D control (3), 7D estrogen (2), 30D control (3), 30D estrogen (4), 90D control (4), 90D estrogen (5), 200D control/control (4), 200D estrogen/control (4), 200D control/estrogen (2), 200D and estrogen/estrogen (4).

Proliferative index was assessed in the epithelial and stromal components in human prostate xenografts (**Fig. 5**). Initial estradiol benzoate-treatment did not affect proliferation in either the epithelial or stromal components of 7, 30, and 90-day xenografts (**Fig. 5A&B**). Epithelial proliferation appears to be increasing from the 7-to 90-day time-points, albeit not significantly (**Fig. 5A**). At 200 days, proliferation index was significantly increased in the epithelial component (7.69± 0.82, n = 4) of the E/E-xenografts compared to C/C controls (**Fig. 5C**), but was still considerably reduced compared to the amount of proliferation seen at the earlier time-points (**Fig. 5A**). No change was found in the 200-day stromal compartment (**Fig. 5D**). Overall percent proliferation in the epithelial compartment across the treatment groups was greater at the earlier time-points (7, 30, and 90-days) with a proliferation index of 10–41% (**Fig. 5A**), compared to epithelial proliferation at 200-days with a proliferation index of 4–8% (**Fig. 5C)**. This demonstrates that the fetal prostate is undergoing epithelial differentiation while adapting to the new rodent host environment as evidenced by epithelial cell proliferation early on in development.

### Estrogen treatment caused gene expression changes involved in ductal growth

Custom PCR arrays were used to determine the expression of 40 prostate genes related to prostate differentiation in 7, 30, and 90-day xenografts. Given the small number of replicates (n = 2–5, with n = 2 for the 7-day estrogen treatment samples only), few statistically significant differences were seen at the earlier time-points. However, estrogen-treated xenografts (“one-hit”) at 90-days exhibited an increase in *KRT8* (1.80, p = 0.002), an epithelial, developmentally-related transcript [35], indicating epithelial compartment maturation (**S2 Table**). This increase in gene expression may also be the result of a glandular compensatory mechanism following early estrogen exposure.

### E/E treatment gene changes are represented in the KEGG prostate cancer pathway

**Fig 6:**
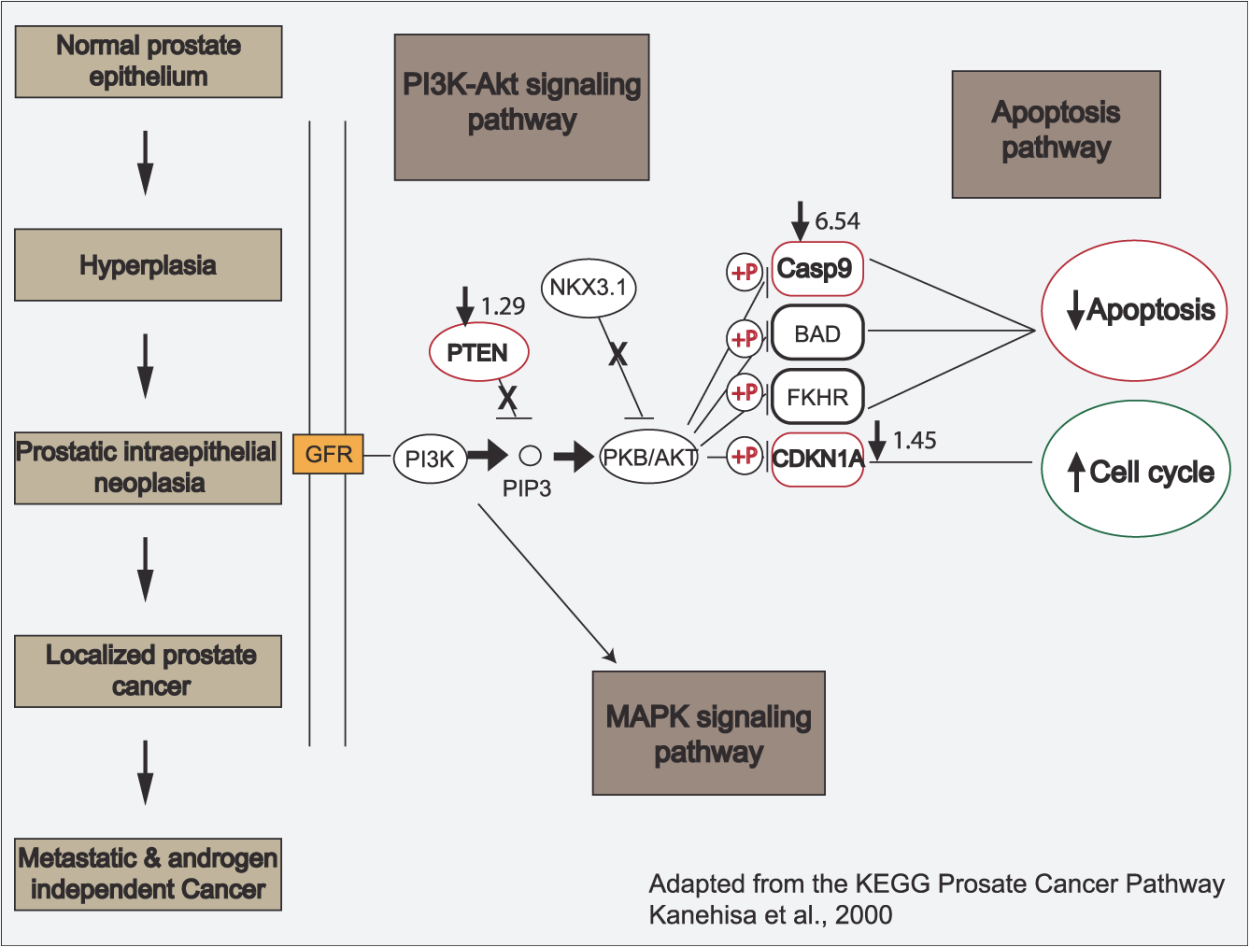
Overview schematic adapted from the KEGG prostate cancer pathway describing the progression of prostate cancer and the events that occur within the cell type coinciding with each step in the xenografts disease transformation. PCR array results from 200-day human xenografts given both an initial and secondary treatment of estrogen demonstrate changes that coincide with the PI3K-Akt signaling pathway, depicting the stage of hyperplasia. This is a transitional stage from normal prostate epithelium to the formation of prostatic intraepithelial neoplasia (PIN) lesions. There is a reduction in the gene expression of the critical tumor suppressor, *PTEN* (−1.29) and *NKX3*.*1* (−2.04, non-significant), and an increase in PKB/Akt that inhibits, via phosphorylation (+P), expression of both *CASP9* (−6.54) and *CDKN1A* (−1.45; also referred to as *p21*). This results in the inhibition of apoptosis, concurrent with an increase in cell cycle progression. Genes circled in red are down-regulated, while those circled in green are up-regulated. Analysis was performed (n = 4, age of human fetal prostate prior to implantation was 16–21.5 weeks gestation) using a LIMMA statistical test, with the circled genes found to be significant (p<0.05).

Xenograft gene expression at 200 days revealed treatment-related effects along the pathway of prostate cancer development (**Table 1**). Two genes, *CASP9* and *CDKN1A*, were down-regulated in every treatment category (E/C, C/E, and E/E), indicating that estrogen treatment may directly affect both apoptosis [36] and cell-cycle progression [37] within the prostate. The greatest changes in gene expression were seen in the E/E treatment group. Significant down-regulation was seen in 6 of the 7 transcripts (*CASP9*, *ESR2*, *CDKN1A*, *CDH1*, *PTEN* and *MYC*), while *TP53* was significantly up-regulated (p-value<0.05). These genes were mapped and evaluated with the KEGG Pathway database [38–40], and three out of the seven altered genes in the 200-day E/E group coincided with morphological changes seen in the transition from normal prostate to prostatic hyperplasia (**Fig. 6**). These gene expression changes occurred in the PI3K-Akt signaling pathway, with an initial decrease in *PTEN* gene expression (−1.29, p = 0.024), a critical tumor suppressor in this pathway [41]. The decrease in *PTEN* gene expression would lead to an increase in PKB/Akt expression, and a subsequent increase in the inhibition, via phosphorylation, of both *CASP9* (−6.54, p = 0.006) and *CDKN1A* (−1.45, p = 0.017). This would ultimately lead to a decrease in apoptosis and an increase in cell-cycle progression, cellular events associated with hyperplasia. In addition, the remaining altered genes that are not depicted in the KEGG Pathway schematic, including *ESR2* (−5.26, p = 0.013) and *CDH1* (−2.08, p = 0.019), are implicated in prostate cancer progression [42, 43]. Interestingly, *MYC* (−1.24, p = 0.052), a promoter of tumorigenesis [44], appears to be slightly down-regulated, while *TP53* (+1.27, p = 0.023), a critical tumor suppressor gene [45], appears to be slightly up-regulated.

**Table 1:** Summary of PCR array results from human prostate xenografts at 200-days post-implantation.

Treatment	Genes	Fold Change (+/−)	P-value	Full gene name	NCBI Gene ID
**Estrogen/Control**	ACTG2	−4.91	0.033	actin, gamma2, smooth muscle, enteric	72
	CASP9	−9.05	0.054	caspase 9, apoptosis-related	842
	CDKN1A	−1.36	0.056	cyclin-dependent kinase inhibitor 1A	1026
**Control/Estrogen**	TP53	1.70	0.003	tumor protein 53	7157
	CASP9	−6.24	0.003	caspase 9, apoptosis-related	842
	CDKN1A	−1.45	0.067	cyclin-dependent kinase inhibitor 1A	1026
**Estrogen/Estrogen**	CASP9	−6.54	0.006	caspase 9, apoptosis-related	842
	ESR2	−5.26	0.013	estrogen receptor beta	2100
	CDKN1A	−1.45	0.017	cyclin-dependent kinase inhibitor 1A	1026
	CDH1	−2.08	0.019	e-cadherin	999
	TP53	1.27	0.023	tumor protein 53	7157
	PTEN	−1.29	0.024	phosphatase and tensin homolog	5728
	MYC	−1.24	0.052	v-myc avian myelocytomatosis viral oncogene homolog	4609

Three different treatment conditions are compared to control xenografts with groups labeled as initial and secondary treatment. Shown are the gene abbreviation, fold change values (+/−), p-value significance, and full gene name (ID). Analysis was performed with linear modeling. Gestational age of human fetal prostate prior to implantation was between 16–21.5 weeks gestation. Sample size (n) for each group is as follows: control/control (4), estrogen/control (4), control/estrogen (2), and estrogen/estrogen (4).

### E/E treatment resulted in stromal CpG methylation

Genome-wide DNA methylation of more than 480,000 CpGs was assessed in 200-day whole prostate xenograft tissue, and in laser-capture microdissected (LCM) epithelial and stromal xenograft tissues. Whole tissue comparisons revealed no significant changes in DNA methylation between two-hit estrogen treatment and control. On the other hand, in LCM-isolated epithelial and stromal compartments, 89 CpG sites were significantly different (q<0.05) between control (C/C) and estradiol benzoate-treated (E/E) samples (**S3 Table**).

**Fig 7:**
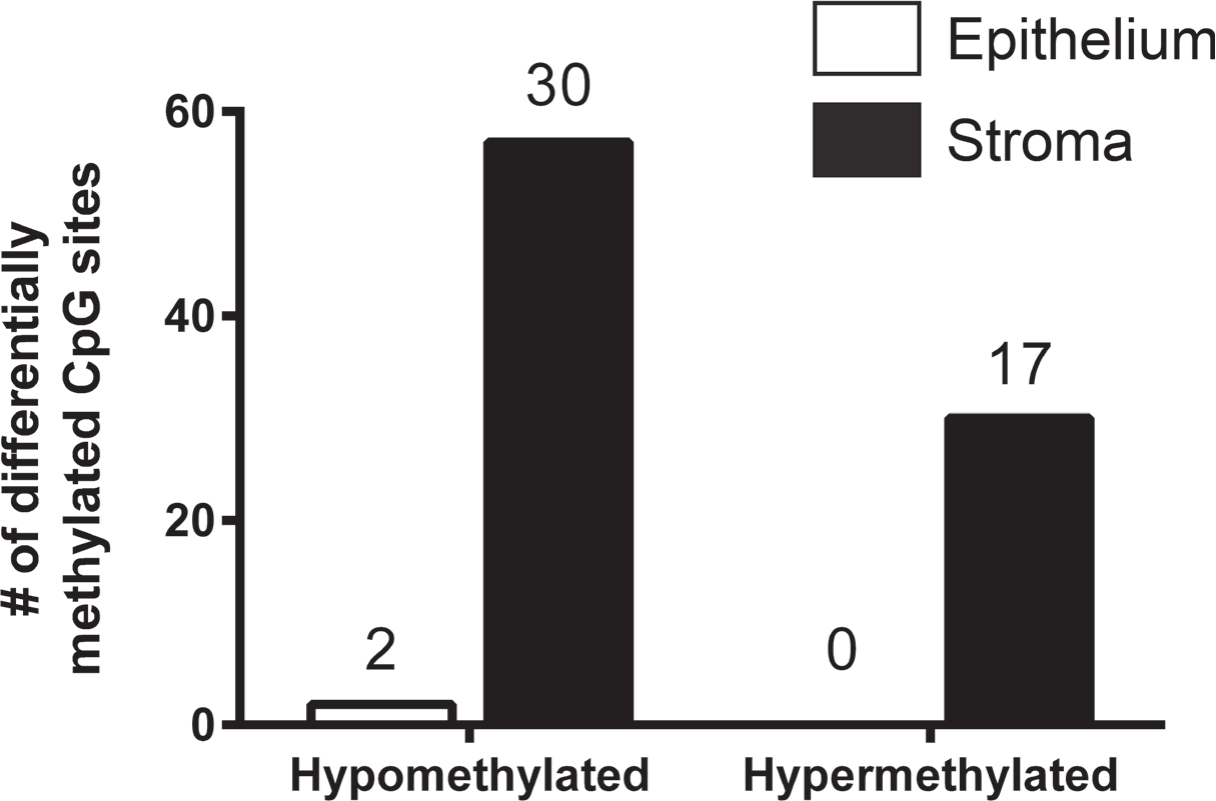
DNA methylation of compartmental epithelial and stromal LCM-microdissected human prostate xenografts. The number of differentially hypomethylated and hypermethylated CpG sites (89 total CpG sites) is shown for the epithelial (white bars) and stromal (black bars) compartments. The number above each bar graph represents the number of genes that correspond to the number of sites found in each group. Stromal hypomethylated (57) CpG sites predominated over epithelial (2) CpG sites. There was no significant epithelial CpG hypermethylation sites (0) found compared to the stromal region (30). Gestational age of human fetal prostate prior to implantation was 16–21.5 weeks old. Sample size (n) for each group is as follows: control/control (3), and estrogen/estrogen (4), with n representing different individual human fetal prostate samples prior to implantation. Significant difference (q<0.05) was seen in 89 CpG sites between control (C/C) and estradiol benzoate-treated (E/E) samples.

The epithelial tissue was significantly hypomethylated at 2 CpG promoter-region sites: *BTD* and one locus whose gene-association is unknown. The stromal compartment had more estrogen-related differential methylation with 57 hypomethylated CpG locations involving 30 genes, and 30 hypermethylated CpG locations involving 17 genes (**Fig. 7**). The most significantly altered genes in stromal hypomethylated CpG locations included *DLX6AS*, *SIM2*, *C7orf44*, *EMX2OS*, *LYRM4*, *LOC145845*, *CACNA1C*, *RAI14*, *SVIL*, and *RCC2*, while altered genes in hypermethylated CpG locations included *CXCL17*, *HRNBP3*, *HOXD12*, *ESRP1*, *HOXD11*, *SRGAP3*, *FOXF2*, *SLC8A2*, *SEMA5B*, and *TBX3* (**Table 2**).

**Table 2:** Stromal-compartment methylation associated gene-changes.

Hypomethylated	Ratio	Hypermethylated	Ratio
DLX6AS	0.41	CXCL17	1.83
SIM2	0.41	HRNBP3	1.89
C7orf44	0.45	HOXD12	2.26
EMX2OS	0.50	ESRP1	2.29
LYRM4	0.50	HOXD11	2.86
LOC145845	0.55	SRGAP3	2.95
CACNA1C	0.59	FOXF2	2.96
RAI14	0.60	SLC8A2	3.51
SVIL	0.62	SEMA5B	3.66
RCC2	0.63	TBX3	4.66

DNA methylation of compartmental epithelial and stromal LCM-microdissected human prostate xenografts. Stromal-compartment list of the top ten methylation-associated gene changes that were hypomethylated or hypermethylated. The ratio value corresponds to the average amount of methylation occurring at each site for the individual gene (Ratio values < 1 indicates hypomethylation; Ratio value > 1 indicates hypermethylation). Gestational age of human fetal prostate prior to implantation was 16–21.5 weeks old, and significant difference of q<0.05 was seen between control (C/C) and estradiol benzoate-treated (E/E) samples. Sample size (n) for each group is as follows: control/control (3), and estrogen/estrogen (4), with n representing different individual human fetal prostate samples prior to implantation.

To further elucidate the predominant canonical pathways affected and how these identified genes clustered by function, the Database for Annotation, Visualization, and Integrated Discovery (DAVID) was used to identify the enriched biological functional groups represented [31]. The results were generated based on significant clustering by gene function, the number of genes, and their enrichment scores, with higher scores having greater relevancy [46]. The full list of gene clusters can be found in **S4 Table**. The top five annotation clusters had enrichment scores between 0.88–2.56 (**Table 3**). The cluster that had the greatest enrichment scores (2.56) included categories for embryonic digit morphogenesis (*TBX3*, *HOXD12*, *LRP5* and *HOXD11*) and developmental protein (*SEMA5B*, *CXCL17*, *WNT5B*, *TBX3*, *HOXD12*, *ROR2*, *LRP5*, *HOXD11* and *SIM2)*. The cluster with the second largest enrichment score (1.28) included 4 genes linked to the Wnt receptor signaling pathway and cell surface receptor linked signal transduction (*WNT5B*, *CSNK1D*, *ROR2*, and *LRP5*). The remaining clusters consisted of functional categories such as cell soma, cell projection, immunoglobulin-like, transmembrane (cell and biological adhesion), ion transport, and plasma membrane.

**Table 3:** Functional gene clusters of 200-day LCM-separated epithelial and stromal human prostate xenograft tissue DNA methylation.

Gene functional classification (based upon significant clusters)	Number of genes (% of clustered genes)	Gene symbols	Enrichment scores
**Annotation Cluster 1:**			**2.56**
Embryonic digit morphogenesis	4 (8.33%)	*TBX3*, *HOXD12*, *LRP5*, *HOXD11*	
Developmental protein	9 (18.75%)	*SEMA5B*, *CXCL17*, *WNT5B*, *TBX3*, *HOXD12*, *ROR2*, *LRP5*, *HOXD11*, *SIM2*	
**Annotation Cluster 2:**			**1.28**
Wnt receptor signaling pathway	4 (8.33%)	*WNT5B*, *CSNK1D*, *ROR2*, *LRP5*	
Cell surface receptor linked signal transduction	4 (8.33%)	*WNT5B*, *CSNK1D*, *ROR2*, *LRP5*	
**Annotation Cluster 3:**			**1.20**
Cell soma	4 (8.33%)	*BTD*, *CACNA1H*, *CACNA1C*, *KALRN*	
Cell projection	5 (10.42%)	*SVIL*, *CACNA1H*, *CACNA1C*, *DNAH8*, *KALRN*	
**Annotation Cluster 4:**			**0.90**
Immunoglobulin-like	4 (8.33%)	*MAG*, *ROR2*, *MCAM*, *KALRN*	
Transmembrane, cell and biological adhesion	3 (6.25%)	*MAG*, *ROR2*, *MCAM*	
**Annotation Cluster 5:**			**0.88**
Ion transport	4 (8.33%)	*SLC8A2*, *CACNA1H*, *CACNA1C*, *KCNG1*	
Plasma membrane	8 (16.67%)	*MAG*, *SVIL*, *CA12*, *CACNA1H*, *ROR2*, *MCAM*, *CACNA1C*, *KCNG1*	

Table was generated using DAVID gene Ontology program and incorporating the 89 differentially methylated CpG-associated genes produced from the Illumina methylation arrays. Table includes the top five generated clusters, number of genes within each category, gene symbols, and their corresponding enrichment scores (range 0.88–2.56). Full gene descriptions and their promoter region CpGs are identified in S3 Table.

## Discussion

Prostate cancer is widely believed to be influenced by a physiological shift in the body’s response to its hormonal milieu. It is possible that while PCa is a disease primarily affecting older men, important underlying hormone triggers originate as early as the *in utero* and young postnatal periods of life [47]. These sensitive periods of development are susceptible to small hormonal changes, and endocrine-disrupting chemicals (EDCs) [3] may alter epigenetic responses, resulting in later-life diseases [7, 48]. While animal models are informative and can provide mechanistic insight, they are not directly representative of the human condition; this is especially true for prostate pathology [49–51]. These differences raise the question of correlation between disruption of the rodent prostate following estrogen exposure and potential hormonal disruption of the human prostate [52]. Our utilization of human fetal prostate tissue xenografts in this study eliminates the need for cross-species extrapolation, allowing us to explore the concept of developmental estrogenization as well as the molecular events that precede later-life prostatic disease, in both whole tissue and LCM-isolated epithelial and stromal compartments.

Communication between the epithelial and stromal compartments in the prostate is critical for proper development and for cross-talk that initiates signals for further differentiation and proliferation in each compartment. For example, epithelial differentiation and growth during the fetal time period is triggered by signals from undifferentiated stromal connective tissue, while signals from the stroma can also influence epithelial growth thereby affecting glandular development [53]. The 30-day control xenografts appear to have acclimated to their mouse host environment, with a great reduction in the amount of basal cell hyperplasia and new development of stromal smooth muscle and fibroblasts (**Fig. 1C**). This is very different from what is observed in the 30-day estrogen-treated xenografts, where basal cell hyperplasia is still prominent (**Fig. 3D)** and the stroma is underdeveloped (**Fig. 1D**). Smooth muscle functions as a support system for glandular tissue, helping to excrete the prostatic secretions [53]. The lack of a proper development in stroma in 30-day estrogen treated xenografts hinders glandular development and maturation and reinforces the presence of small, rudimentary glands that appear with little to no secretory activity within the lumen. Xenografts appear phenotypically similar by 90-days, containing both proper ductal and stromal components (**Fig. 1E&F**). This suggests that there are molecular compensatory events occurring between day 30 and 90 that allow recovery from estrogen exposure in the human prostate tissue.

To investigate if there were persistent estrogenic effects following an initial estrogen exposure, a subset of animals (day 90) received a secondary estrogen plus testosterone exposure. These combined initial plus secondary estrogen-exposed xenografts (E/E-samples) revealed marked glandular hyperplasia and a significant amount of PSA expression (Figs. **2D, 2H, and 3F**) with no evidence of PIN lesions or further progression along the prostate carcinogenic sequence. This result sharply contrasts with previous findings in rat models showing that both low and high doses of estradiol result in PIN. In addition, if a rodent neonatal exposure to high-dose estradiol preceded a secondary exposure to estradiol and testosterone, then the incidence of PIN lesions increased from 40% to 100% [10, 11, 14]. This markedly different response suggests that human tissue is more resistant to the early life carcinogenic effects of estrogen exposure compared to rat models, albeit through unknown mechanisms, or alternatively that effects in humans take much longer to present. It is interesting that while estrogen levels are significantly increased in the 7-day estrogen treatment groups, as well as in the 200-day C/E and E/E groups, that this dose of estradiol may not be sufficient enough to induce cancerous lesions (**S2 Fig.)**. It is also noteworthy that there are clear differences in the susceptibility of different strains of rats to hormonal carcinogens and also marked differences between rats and mice, with rats being a markedly more susceptible model, so the fact that human samples did not go on to form premalignant lesions in the available time window does not preclude the possibility that such response could not ultimately occur.

To determine whether the effects of estrogen were persistent following an initial treatment, we evaluated gene expression changes at 200 days (**Table 1**). In addition to having the greatest histological changes, E/E treatment led to the highest number of individual gene changes compared to other treatment groups. Our results showed that PI3K-Akt signaling pathway was primarily affected, with changes in *PTEN*, *Casp9*, and *CDKN1A*, consistent with reducing apoptosis and increasing cell cycle progression (**Fig. 6**). This corresponds to the pathological manifestation of the intermediate transitory stage of hyperplasia, which was observed in these samples by histopathology.

Epigenetic mechanisms, such as DNA methylation, have been proposed to play a pivotal role in maintaining the persistent effects that are seen in adult rodents following neonatal administration of estrogen [13]. DNA methylation has been favored as a primary mechanism because it is a process in which heritable, stable changes (e.g. gene activation or silencing) are induced through the addition of a methyl group to the 5’carbon of a CpG cytosine of DNA [48, 54, 55]. Hypermethylation of promoters, often leading to silencing of genes, has been implicated as a way to halt damage repair systems, and engage cell cycle progression to promote metastasis of cancerous cells [56]. This study used tissue-specific LCM of 200-day C/C and E/E xenografts to examine DNA methylation, which differs from our gene expression data that evaluated whole prostate xenograft tissue and found changes within the PI3K-Akt pathway. The genes that were discovered to be significant in our gene array data were also evaluated in our DNA methylation results but were not found to be significant. LCM was not performed on our gene expression data due to the limited amount of tissue obtained in this particular study, but is being pursued in future studies. This interesting finding demonstrated that LCM is an important tool to investigate compartmental specific changes. The importance of LCM as a tool used in our study was able to reveal that in our data stromal tissue was most affected by estrogen exposure, with 87 significantly altered CpG sites, compared to 2 CpG sites significantly altered in the epithelium (**Fig. 7**).

Many studies focus on epithelial-stromal communication in prostate carcinogenesis, but it may be that the stroma itself plays a much larger role, compared to the epithelium, in maintaining the homeostasis of the prostate [53, 57]. The stroma contains many different components, including immune cells, smooth muscle, vasculature, fat, fibroblasts, and nerves that all need to work together for normal tissue development and adult growth quiescence [58]. Some components of the stroma, including smooth muscle, express estrogen receptors and may be a primary target of endocrine disrupting chemicals [59]. One study in rodents demonstrated that in prostate tissue recombinants of epithelium and stroma, treatment using testosterone and estrogen can induce prostate carcinogenesis; however it is the stromal microenvironment that confers on the epithelial cells to a cancerous phenotype [60]. In addition, carcinogenesis is driven by androgen receptors located in the stroma [61]. The data presented in this study emphasizes the importance of the stroma as a potential initiator of prostate disease, and provides evidence that estrogen primarily targets the stroma.

A closer examination of the top ten gene-associated CpGs that were hypomethylated and hypermethylated in the stromal compartment (**Table 2**) revealed changes that correspond to a proclivity towards prostate disease progression. In this study, Single-Minded Homologue 2 (SIM2) was hypomethylated in 200-day xenografts that were given E/E treatment. The expression of SIM2, a transcription factor, is increased in prostate cancer [62, 63] and its hypomethylation status (ratio 0.41) in stromal tissue provides evidence that SIM2 transcription is upregulated. Additionally, Forkhead Box 2 (FOXF2) is a transcription factor that is important for many cellular functions and its expression is decreased in prostate cancer [64, 65]. This coincides with our findings that FOXF2 is significantly hypermethylated (ratio 2.96) in the stromal compartment of 200-day E/E-treated xenografts indicating that FOXF2 transcription is repressed following estrogen exposure. Taken together, these findings correspond to the notion that E/E-exposure affects the genes that are involved in PCa development.

Our data support the conclusion that estrogen treatment primarily affects the stroma, a fundamental player in the development of prostatic disease formation. We have identified the top gene-associated CpGs that are hypo- or hyper-methylated in response to the “one-hit” versus “two-hit” model, and these genes will be pursued in future mechanistic studies of developmental estrogenic exposures. This is the first time that these genes have been identified as a result of estrogen-induced damage within the human prostate, and contributes new avenues of research in this ever-growing field of prostate research.

Previous animal research has laid the foundation for studying the developmental basis of adult disease, but the use of human tissue is largely unexplored. This leads to the important question of whether the results obtained in animal studies truly represent what human processes. While this model has numerous benefits, there are several limitations that are inevitable in the study of human fetal tissue.

While the use of human tissue confers many important advantages of human PCa, it is not without its own limitations. For example, there are potential problems with the transplanted tissue, which is obtained from spontaneous pregnancy losses. The most common cause of still-birth is chromosomal anomaly that may affect prostate tissue, confounding cytogenetic evaluations. In addition, the incidence of these pregnancy losses and our ability to procure tissue is unpredictable, and the range of gestational age is wide, both causing problems in the study design since estrogen can have different effects during various phases of development. Finally, use of a rodent host is necessarily limited by the lifespan of the animal; because it is relatively short, we have not yet been able to study human tissue long enough for it to develop into the final stages of prostate cancer. The limited amount of tissue that is obtained from the experiments can also prevent some studies from being performed, and requires the researcher to prioritize which endpoints are most desired within the study design.

In spite of these issues, the benefits of studying human tissue overcome the limitations. The information gathered from these studies does not require species extrapolation, and can provide new targets of investigation in the field of PCa therapy.

## Conclusions

The present study provides evidence of species-specific differences in the sensitivity of fetal prostate to estrogen exposure. The human fetal prostate is more resistant to estrogenic exposures compared to previous studies performed in rats. Early estrogen exposure alone affects branching morphogenesis, leading to the presence of rudimentary glands, and hinders basal cell communication. These estrogenic effects are persistent and present in a 200-day differentiated adult gland. A closer look at the gene changes following an initial and a secondary estrogenic exposure revealed changes in the PI3K-Akt pathway associated with a hyperplastic tissue response. Estrogen exposure-related alterations in DNA methylation primarily occurred in the stroma, emphasizing the role of this compartment in carcinogenesis. This human fetal xenograft model has provides new avenues for exploring the prostate carcinogenic sequence, and underscores the importance of using human tissues to study early life estrogen exposures of the prostate.

## Supporting Information

S1 Fig Dosing paradigm of early and later-life effects of estrogenic exposures. This model is based upon one examining the fetal basis of adult prostatic disease in rodents. Human fetal prostates (gestational age 12–24 weeks) were received from spontaneous pregnancy losses (day 1), and implanted into the renal subcapsular space of an immunodeficient athymic nude mouse host. Immediately following xenograft surgery, mice were treated with either a subcutaneous injection of corn oil (control) or 250 μg/kg of β-estradiol 3-benzoate (treatment) in corn oil on days 1, 3, and 5 for a total of 3 injections. A subset of mouse hosts was collected on 7, 30, and 90 days, representing a “one-hit” exposure to examine the effects of early estrogenic treatment. An additional subset of animals received a secondary treatment of 2.5-mg β-estradiol 3-benzoate (slow release pellet) and a silastic capsule of 25 mg testosterone on day 90, for a period of 110 days. Animals that received both initial and secondary treatments (“two-hit”) were collected on day 200 to evaluate the effects of early and long-term estrogen exposure.
(TIF)

S2 Fig Serum hormone measurements of luteinizing hormone (LH), follicular stimulating hormone (FSH), testosterone, and estradiol in mouse hosts given either an (A-D) initial treatment of corn oil or estrogen, (E-H) or 200-day hosts given initial and secondary treatments of placebo or estrogen. An initial exposure of estrogen does not affect the serum levels of (**A**) LH, (**B**) FSH, or (**C**) testosterone at 7, 30, or 90 days. (**D**) The level of estradiol (pg/ml) is significantly increased in 7-day xenografts. (**G**) A later-life exposure to estrogen had no effects on testosterone levels, since testosterone was co-administered, but led to a significant decrease in both (**E**) LH and (**F**) FSH levels, as well as, a significant (**H**) increase in estradiol levels at the 200-day time-point. Lines and bars indicate the mean concentration ± SEM. Legend: —●— Control, -■- Estrogen. Significant difference is from the respective control; *** indicates p<0.001 as compared by a two-way ANOVA with a Bonferonni correction (**D**); ** indicates p<0.01, **** p<0.0001 as compared by a one-way ANOVA with a Bonferonni correction (**E, F, &H**). The X-axis on the 200-day bar graphs (**E-H**) are depicted as initial/secondary treatment in which C = control, and E = estrogen treatment.
(TIF)

S1 Table Full list of genes selected for the custom SABiosciences PCR Array. (TIF)

S2 Table Summary of PCR array results from human prostate xenografts given an initial exposure of estradiol and collected at 7, 30 and 90 days post-implantation. Analysis was performed using a LIMMA statistical test (p-value significance).
(TIF)

S3 Table Full list of genes and their promoter region CpG sites associated with epithelial and stromal differential methylation in 200-day E/E-treated xenografts. (XLS)

S4 Table List of functional genes clusters generated by DAVID in 200-day E/E-treated xenografts. (XLS)
