# Supplementary figures and images for: Developmental Exposure to Estrogen Alters Differentiation and Epigenetic Programming in a Human Fetal Prostate Xenograft Model
Source: PLoS One. 2015 Mar 23;10(3):e0122290. doi: 10.1371/journal.pone.0122290 (PMC4370592; doi:10.1371/journal.pone.0122290)

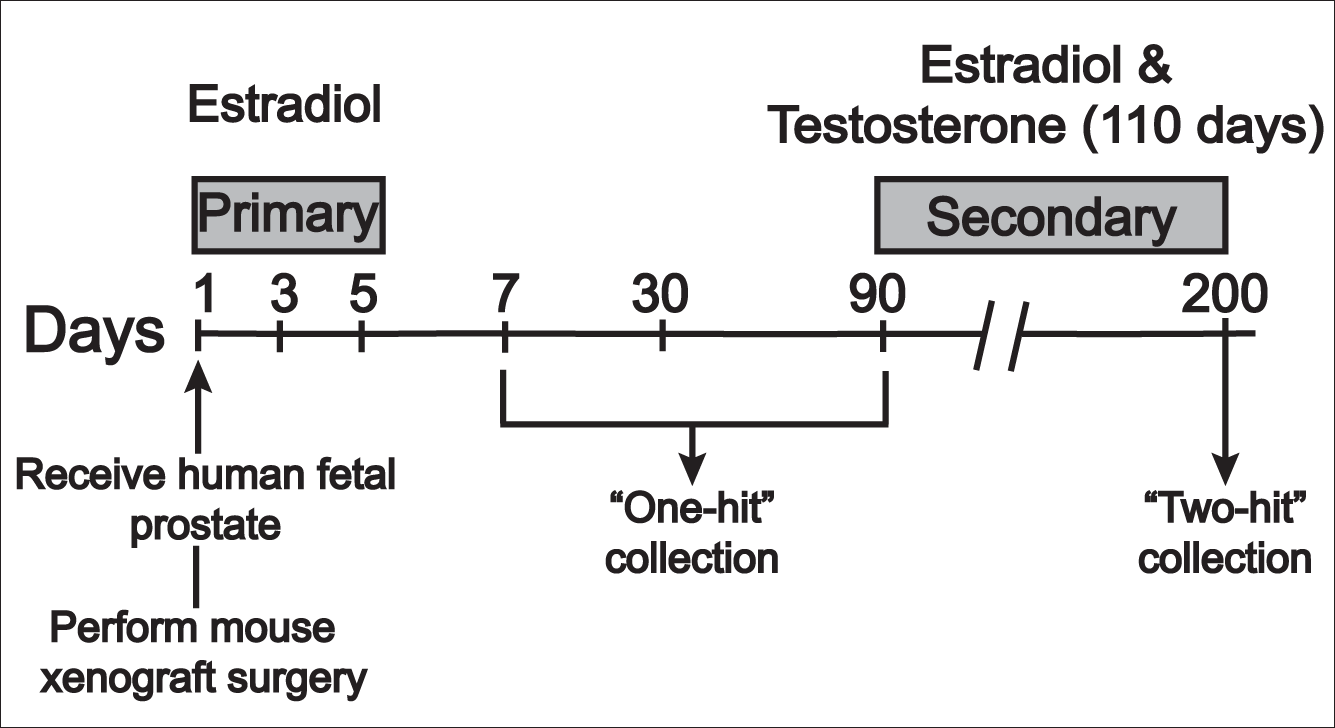

Supplement: S1 Fig — This model is based upon one examining the fetal basis of adult prostatic disease in rodents. Human fetal prostates (gestational age 12–24 weeks) were received from spontaneous pregnancy losses (day 1), and implanted into the renal subcapsular space of an immunodeficient athymic nude mouse host. Immediately following xenograft surgery, mice were treated with either a subcutaneous injection of corn oil (control) or 250 μg/kg of β-estradiol 3-benzoate (treatment) in corn oil on days 1, 3, and 5 for a total of 3 injections. A subset of mouse hosts was collected on 7, 30, and 90 days, representing a “one-hit” exposure to examine the effects of early estrogenic treatment. An additional subset of animals received a secondary treatment of 2.5-mg β-estradiol 3-benzoate (slow release pellet) and a silastic capsule of 25 mg testosterone on day 90, for a period of 110 days. Animals that received both initial and secondary treatments (“two-hit”) were collected on day 200 to evaluate the effects of early and long-term estrogen exposure. (TIF) [file pone.0122290.s001.tif]

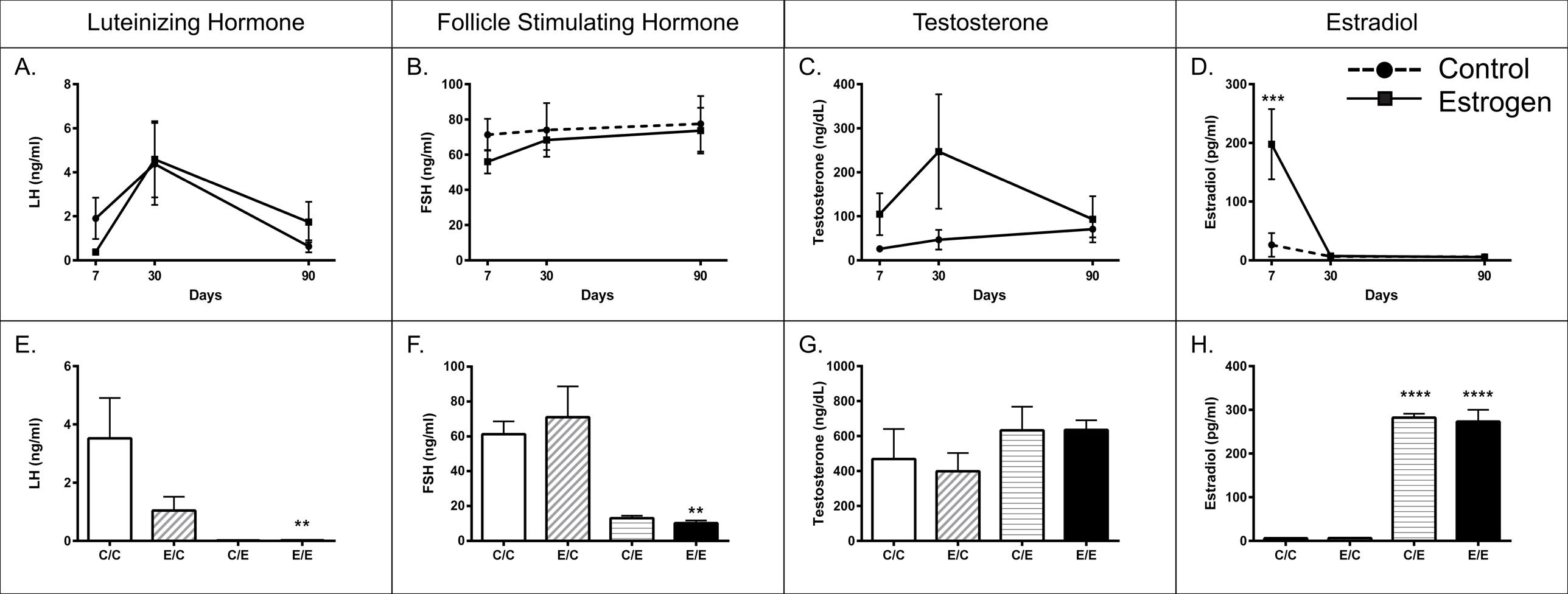

Supplement: S2 Fig — An initial exposure of estrogen does not affect the serum levels of (A) LH, (B) FSH, or (C) testosterone at 7, 30, or 90 days. (D) The level of estradiol (pg/ml) is significantly increased in 7-day xenografts. (G) A later-life exposure to estrogen had no effects on testosterone levels, since testosterone was co-administered, but led to a significant decrease in both (E) LH and (F) FSH levels, as well as, a significant (H) increase in estradiol levels at the 200-day time-point. Lines and bars indicate the mean concentration ± SEM. Legend: —●— Control, -■- Estrogen. Significant difference is from the respective control; *** indicates p<0.001 as compared by a two-way ANOVA with a Bonferonni correction (D); ** indicates p<0.01, **** p<0.0001 as compared by a one-way ANOVA with a Bonferonni correction (E, F, &H). The X-axis on the 200-day bar graphs (E-H) are depicted as initial/secondary treatment in which C = control, and E = estrogen treatment. (TIF) [file pone.0122290.s002.tif]

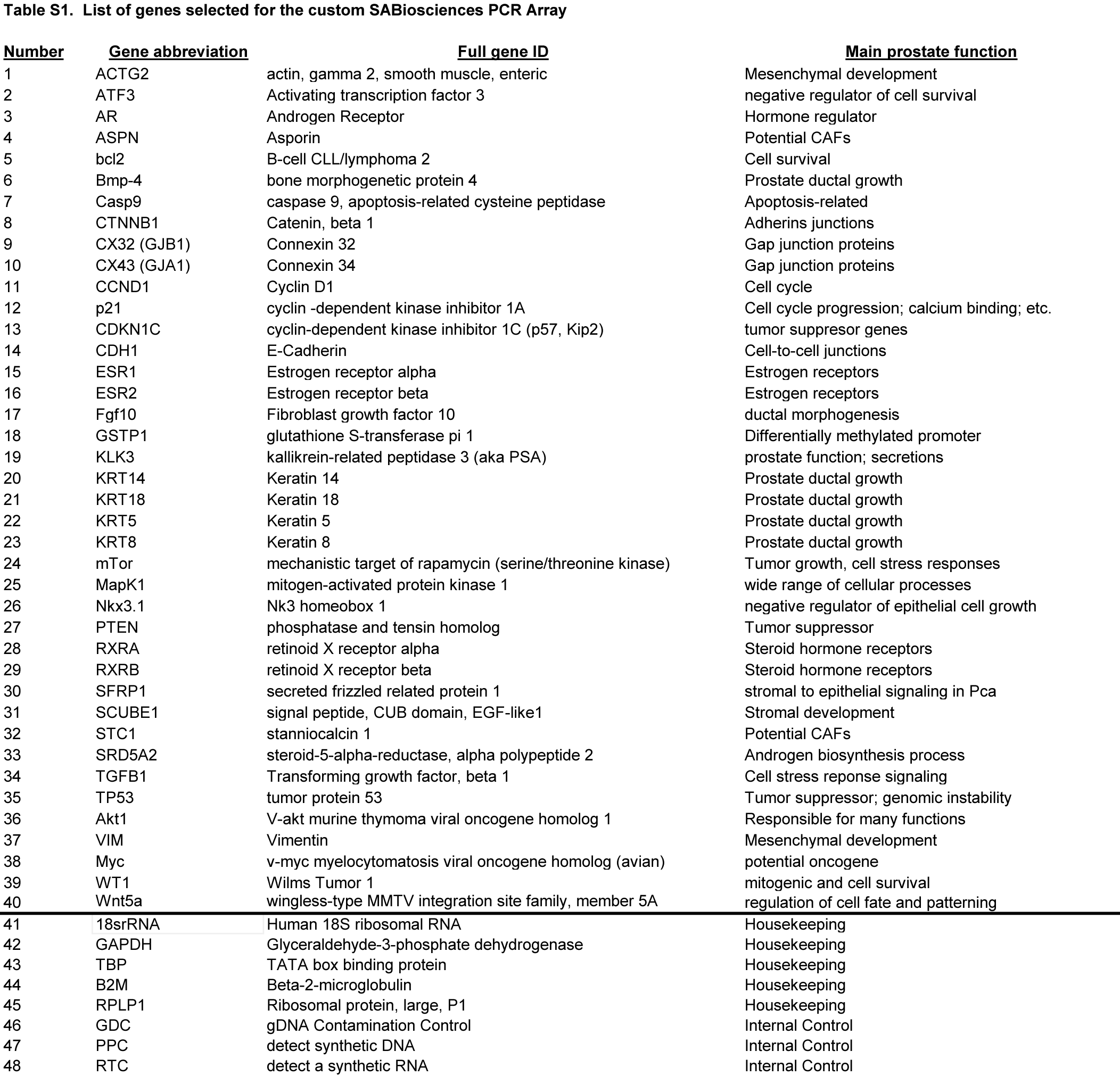

Supplement: S1 Table — (TIF) [file pone.0122290.s003.tif]

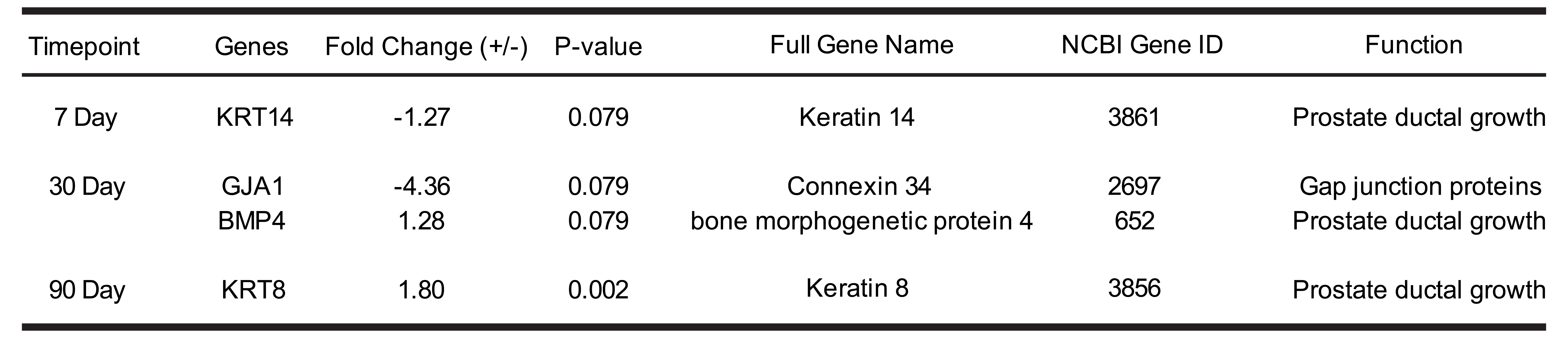

Supplement: S2 Table — Analysis was performed using a LIMMA statistical test (p-value significance). (TIF) [file pone.0122290.s004.tif]
